# LNRRIL6, a novel long noncoding RNA, protects colorectal cancer cells by activating the IL‐6–STAT3 pathway

**DOI:** 10.1002/1878-0261.12538

**Published:** 2019-09-30

**Authors:** Jiaxing Wang, Junfeng Zhou, Caiyun Jiang, Jing Zheng, Hiroki Namba, Pan Chi, Tetsuya Asakawa

**Affiliations:** ^1^ Department of Gastrointestinal Surgery The First Affiliated Hospital of Fujian Medical University Fuzhou China; ^2^ Department of Colorectal Surgery Fujian Medical University Union Hospital Fuzhou China; ^3^ Fujian Institute of Hematology Fujian Provincial Key Laboratory on Hematology Fujian Medical University Union Hospital Fuzhou China; ^4^ Department of Neurosurgery Hamamatsu University School of Medicine Hamamatsu Japan; ^5^ Research Base of Traditional Chinese Medicine Syndrome Fujian University of Traditional Chinese Medicine Fuzhou China

**Keywords:** colorectal cancer, IL‐6, IL‐6−STAT3 pathway, LNRRIL6, long noncoding RNA

## Abstract

Long noncoding RNAs (lncRNAs) are emerging as critical regulators of cancer. There is a comparable number of lncRNAs to protein‐coding genes, but the expression patterns, functions, and molecular mechanisms of most lncRNAs in colorectal cancer (CRC) remain unclear. In this study, we report the identification of a novel lncRNA, named long noncoding RNA regulating IL‐6 transcription (LNRRIL6), which is upregulated in CRC tissues and cell lines. Increased LNRRIL6 expression is associated with aggressive clinicopathological characteristics and poor prognosis of CRC patients. Functional experiments showed that enhanced expression of LNRRIL6 promotes CRC cell proliferation and survival *in vitro* and CRC tumor growth *in vivo*. Conversely, depletion of LNRRIL6 inhibits CRC cell proliferation and survival *in vitro* and CRC tumor growth *in vivo*. Mechanistically, we revealed that LNRRIL6 physically binds to the IL‐6 promoter, thereby increasing IL‐6 transcription, inducing IL‐6 autocrine signaling, and activating the IL‐6/STAT3 pathway. The expression of IL‐6 is positively associated with that of LNRRIL6 in CRC tissues. Blocking the IL‐6/STAT3 pathway using the FDA‐approved IL‐6‐receptor antagonist antibody, tocilizumab, abolished the oncogenic role of LNRRIL6 in CRC. Taken together, these findings identify a novel lncRNA, LNRRIL6, that promotes CRC cell survival through activation of the IL‐6/STAT3 pathway and suggest that LNRRIL6 may be a potential prognostic biomarker and therapeutic target for CRC.

**Abbreviations**
BLAST: Basic Local Alignment Search Tool
CCAL: CRC‐associated LncRNA
ChIRP: chromatin isolation by RNA purification
CRC: colorectal cancer
EdU: ethynyl deoxyuridine
ELISA: enzyme‐linked immunosorbent assay
GPRC5A: G protein‐coupled receptor class C group 5 member A
IL‐6: interleukin 6
LncRNAs: long noncoding RNAs
LNRRIL6: long noncoding RNA regulating IL‐6 transcription
STAT3: signal transducer and activator of transcription 3
TUNEL: terminal deoxynucleotidyl transferase (TdT)‐mediated dUTP nick end labeling

## 1 Introduction

Colorectal cancer (CRC) is the third most common cancer and the fourth leading cause of cancer‐related mortality worldwide; thus, it has long been a significant public health concern (Torre *et al*., 2015). Despite the availability of surgical intervention in early stages, the CRC outcome remains poor due to high metastatic potential. Hence, a more comprehensive understanding of the cellular behavior and molecular mechanisms underlying the carcinogenesis and progression of CRC is warranted to identify novel targets and therapeutics.

Several non‐protein‐coding regions of the human genome are involved in the initiation and progression of CRC (Chen *et al*., 2016; Distaso *et al*., 2008; Rui *et al*., 2015), a majority of which are transcribed into noncoding RNAs (ncRNAs). Long noncoding RNAs (lncRNAs) are longer than 200 nucleotides and are a critical component of ncRNAs. Mounting evidence has demonstrated that dysregulation of lncRNAs may play a role in the pathophysiological processes of cancer (Li *et al*., 2017a; Lin *et al*., 2016; Liu *et al*., 2016; Tong *et al*., 2015), including CRC. LncRNAs, such as CCAL, PINCR, LINC01133, SNHG5, loc285194, CCAT2, and MALAT1, can modulate the biological behaviors of CRC cells (Chaudhary *et al*., 2017; Damas *et al*., 2016; Kong *et al*., 2016; Li *et al*., 2017b; Ling *et al*., 2013; Liu *et al*., 2013; Ma *et al*., 2016). However, the roles of most lncRNAs in CRC remain unclear due to the extensive volume of lncRNAs (Yang *et al*., 2016; Ye *et al*., 2015). A recent study defined the CRC‐associated lncRNA (CCAL) and established its critical role as a regulator of tumorigenesis and CRC progression (Ma *et al*., 2016), suggesting that an investigation of mechanisms related to lncRNAs may open new avenues for exploring novel therapeutic targets. In our pre‐experimental attempt to identify the target lncRNA, we performed GeneChip screening based on microarray results obtained from a previous study (Inamura *et al*., 2008). We observed a significant difference in the expression of lncRNAs between tumor and normal tissues. Among several lncRNAs, the novel lncRNA, AK024522 (probe number: ASLNC17018; located at chromosome 12p13.1; 2017 nucleotides in length), was considered after searching on the Basic Local Alignment Search Tool (BLAST). Intriguingly, we identified a significant AK024522 binding locus on the interleukin‐6 (IL‐6) promoter spanning 870–1180 bp (Fig. S1). The presence of 83% identities between AK024522 and the IL‐6 promoter suggested that AK024522 directly binds to the IL‐6 promoter; thus, we named this lncRNA long noncoding RNA regulating IL‐6 transcription (LNRRIL6). LNRRIL6 is located on the intron of G protein‐coupled receptor class C group 5 member A (GPRC5A). More precisely, it is located in a DNA region enriched with H3K27Ac histone modifications, as reported by the Encode project (UCSC genome browser), suggesting it may be a potential enhancer. Several studies have described that GPRC5A plays a role in tumor initiation by affecting the signal transducer and activator of the transcription 3 (STAT3) signaling pathway (Chen *et al*., 2010; Jahny *et al*., 2017; Liu *et al*., 2017; Zhong *et al*., 2015).

The IL‐6–STAT3 signaling pathway plays a crucial role in several cancers, including CRC (Johnson *et al*., 2016; Yeh *et al*., 2016). We hypothesized that LNRRIL6 may serve as an IL‐6 promoter through the IL‐6–STAT3 signaling pathway and protect CRC cells. Hence, this study aims to validate the protective effects of LNRRIL6 on CRC and to provide concrete evidence of these protective effects at a clinical, tissue, and cellular level. In addition, we investigate potential mechanisms associated with LNRRIL6 and the IL‐6–STAT3 signaling pathway, crucial for determining the potential use of LNRRIL6 as a novel therapeutic target for CRC.

## 2 Materials and methods

### 2.1 Experimental design and ethical considerations

The experimental design for obtaining concrete evidence for the protective effect of LNRRIL6 on CRC cells is shown in Fig. S2. The validation comprised of the following three levels: (a) clinical, where we compared the clinical outcomes of patients with high and low LNRRIL6 expression; (b) tissue, where we observed the distribution of LNRRIL6 in normal and tumor tissues, followed by investigating IL‐6 mRNA expression in tumor tissues and its correlation with IL‐6; and (c) cellular, where we validated the effects of LNRRIL6 on CRC cells and compared results with a normal human colon mucosal epithelial cell line (NCM460) *in vitro* and *in vivo* in both LNRRIL6 high‐expression cells and LNRRIL6 knockdown cells. In addition, we investigated IL‐6–STAT3‐related mechanisms using an IL‐6 receptor antagonist, tocilizumab. All experiments were replicated three times, and continuous variables were averaged.

From January 2012 to December 2012, we enrolled 66 patients who were pathologically diagnosed with CRC at the Fujian Medical University Union Hospital (Fuzhou, China). Patients older than 18 years and those who had undergone R0 resection were eligible for the study; however, those with metastasis, a concurrent diagnosis of familial adenomatous polyposis, Lynch syndrome or irritable bowel disease (IBD), or metachronous or synchronous CRC were excluded from the analysis. In this study, all patients underwent resection, and their tissue samples were diagnosed by histopathological examination. In addition, adjacent normal mucosal tissues were obtained as control. Patients were followed up for 60 months after resection and examined for clinical outcomes. This study was designed and performed as per the guidelines of the Declaration of Helsinki of the World Medical Association (2000) and was approved and supervised by the Ethics Committee of the Fujian Medical University (approval No: 2017KY073). We obtained written informed consent from all patients or their relatives after a comprehensive explanation of the study protocol.

For *in vivo* experiments, we used the flanks of 20 female athymic nude mice (6–8 weeks old; body weight, 15–25 g; SLRC Laboratory Animal Center, Shanghai, China). Of note, all animals were carefully treated as per the National Institute of Health Guidelines for the Care and Use of Laboratory Animals. In addition, all experimental procedures were approved by the Animal Care and Use Committee of the Fujian Medical University (authorization No: 2017KY073).

### 2.2 LNRRIL6 expression in tissues and patients with CRC

The general and clinical information of each patient in this study was collected (Table 1). Next, we examined the expression of LNRRIL6 in CRC tissues and adjacent normal mucosal tissues using quantitative real‐time polymerase chain reaction (qRT–PCR). The freshly resected tissue samples were snap‐frozen in liquid nitrogen and stored at −80 °C for further use in qRT–PCR.

**Table 1:** A correlation between LNRRIL6 expression and clinicopathological characteristics in patients with CRC

Characteristics	LNRRIL6 expression		*P*
	Low (*n* = 33)	High (*n* = 33)	
Gender			
Male	19	18	0.804
Female	14	15	
Age (years)			
< 60	13	16	0.457
≥ 60	20	17	
Tumor location			
Colon	16	16	1.000
Rectum	17	17	
Tumor size (cm^3^)			
< 5	20	12	0.049^a^
≥ 5	13	21	
Differentiation			
Well‐moderate	23	14	0.026^a^
Poor	10	19	
Depth of invasion			
T1 + T2	20	13	0.085
T3 + T4	13	20	
Lymph node metastasis			
Yes	7	15	0.037^a^
No	26	18	

a Represents *P* < 0.05.

The expression of LNRRIL6 in CRC cell lines was investigated. We obtained the human colon normal epithelial cell line (NCM460) and CRC cell lines (HCT‐116, LoVo, SW480, SW620, and HT‐29) from the Cell Bank of the Institute of Biochemistry and Cell Biology of the Chinese Academy of Sciences (Shanghai, China). NCM460 was maintained in Dulbecco's modified Eagle's medium (Invitrogen, Carlsbad, CA, USA), HCT‐116 and HT‐29 were maintained in McCoy's 5A medium (Sigma‐Aldrich, St. Louis, MO, USA), LoVo was maintained in Ham's F‐12K medium (Invitrogen), and SW480 and SW620 were maintained in L‐15 medium (Invitrogen). All cells were cultured in a medium supplemented with 10% fetal bovine serum (Invitrogen) in a humidified incubator containing 5% CO_2_ at 37 °C. Where indicated, cells were treated with 25 ng/mL doxorubicin (Selleck, Houston, TX, uSA) for 24 h or 5 μg·mL^−1^ tocilizumab (Genentech, South San Francisco, CA, USA) for the indicated time. All cells were cultured for use in qRT–PCR.

Total RNA was isolated using the TRIzol reagent (Invitrogen) as per the manufacturer's protocol, followed by treatment with DNase I to remove DNA. Subsequently, the isolated RNA was used to perform reverse transcription with M‐MLV Reverse Transcriptase (Invitrogen) as per the manufacturer's instructions. Next, we performed qRT–PCR using SYBR^®^ Premix Ex Taq™ II (Takara, Dalian, China) on the ABI StepOnePlus Real‐Time PCR System (Applied Biosystems, Foster City, CA, USA) as per the manufacturer's instructions. β‐Actin was used as an endogenous control for quantification of RNA expression, which was calculated with the comparative *C*_t_ method. The gene‐specific primer sequences used were as follows: LNRRIL6, 5′‐CACTAAGATAGGAGCGGGG‐3′ (forward) and 5′‐TGAGACAGGGTAGGATGGG‐3′ (reverse); IL‐6, 5′‐GAGTAGTGAGGAACAAGCC‐3′ (forward) and 5′‐CAACAACAATCTGAGGTGC‐3′ (reverse); CDC25A, 5′‐GCGTGTCATTGTTGTGTTTC‐3′ (forward) and 5′‐GGTAGTGGAGTTTGGGGTA‐3′ (reverse); cyclin D1, 5′‐TCCTCTCCAAAATGCCAGAG‐3′ (forward) and 5′‐GGCGGATTGGAAATGAACTT‐3′ (reverse); survivin, 5′‐GCAGCCCTTTCTCAAGGACC‐3′ (forward) and 5′‐AGTGGATGAAGCCAGCCTCG‐3′ (reverse); BCL2, 5′‐CTTCGCCGAGATGTCCAG‐3′ (forward) and 5′‐CCCAGCCTCCGTTATCCT‐3′ (reverse); and β‐actin, 5′‐GGGAAATCGTGCGTGACATTAAG‐3′ (forward) and 5′‐TGTGTTGGCGTACAGGTCTTTG‐3′ (reverse). In addition, we performed 5′‐RACE and 3′‐RACE assays to determine the transcriptional initiation and termination sites of LNRRIL6 with a 5′/3′ RACE Kit (Roche, Mannheim, Germany) as per the manufacturer's instructions. The gene‐specific primer sequences used for the PCR of RACE assays were as follows: SP1, 5′‐TTCTTTCTCCCCCTCATCC‐3′; SP2, 5′‐TGTGATGAGTCTCCCGCTT‐3′; SP3, 5′‐ACGCAGTCCTTCCCAGAGCATC‐3′; and SP5, 5′‐GCATCAGGTGCTAGGCAAGTG‐3′.

In this study, the cutoff value for high and low expression of LNRRIL6 was defined by assessing the heterogeneity of LNRRIL6 expression in both tumor and normal tissues with a log‐rank test statistical analysis, as previously described (Cai *et al*., 2014). Hence, in the present study, the range of the low‐expression group was 1.629238–7.73354 and that of the high‐expression group was 8.515294–50.86561.

### 2.3 Correlation between LNRRIL6 and the proliferation and survival of CRC cells

#### 2.3.1 Establishment of LNRRIL6 overexpressing cells and LNRRIL6 knockdown cell models

Full‐length sequences of LNRRIL6 were cloned into the lentiviral vector pLV6/EF‐1a/Puro (GenePharma, Shanghai, China) in HCT‐116 and LoVo cells to establish LNRRIL6 overexpressing cells. The empty vector, pLV6/EF‐1a/Puro, was used as a negative control. For LNRRIL6 knockdown cells, two pairs of cDNA oligonucleotides targeting LNRRIL6 were subcloned into the SuperSilencing shRNA expression vector pGLVU6/Puro (GenePharma). The target sites were 5′‐TGGGAGTAAAGGGAAAGAGTT‐3′ (sh1) and 5′‐GGTTTAGTCACTGAGTACTGG‐3′ (sh2). A scrambled nontargeting sequence was used as a negative control. To produce lentiviruses for LNRRIL6 overexpression or silencing, the vectors described above were cotransfected with pGag/Pol, pRev, and pVSV‐G (GenePharma) into HEK‐293T cells with Lipofectamine 3000 (Invitrogen) as per the manufacturer's instructions. Infectious lentiviruses were harvested after 72 h transfection, filtered, and concentrated using Lenti‐X Concentrator (Clontech, Palo Alto, CA, USA). For the stable transfection of LNRRIL6 in CRC cells, HCT‐116 and LoVo cells were transfected with 2 × 10^6^ transducing units of LNRRIL6 overexpression or silencing lentiviruses and selected with 1 μg·mL^−1^ puromycin for 4 weeks.

#### 2.3.2 Cell proliferation and apoptosis assays

Cell proliferation was evaluated using the Glo cell viability assay and the ethynyl deoxyuridine (EdU) incorporation assay. For the Glo cell viability assay, 3000 CRC cells were seeded into 96‐well plates and cultured for the indicated time. At each time point, the luminescence values were measured using the CellTiter‐Glo^®^ Luminescent Cell Viability Assay (Promega, Madison, WI, USA), following the manufacturer's protocol. In addition, the EdU incorporation assay was performed using the EdU kit (Roche) as per the manufacturer's instructions. Furthermore, all results were collected using the Zeiss fluorescence photomicroscope (Carl Zeiss, Oberkochen, Germany) and quantified by counting 10 random fields.

We evaluated cell apoptosis using the terminal deoxynucleotidyl transferase (TdT)‐mediated dUTP nick end labeling (TUNEL) assay, which was performed using the In Situ Cell Death Detection Kit (Roche) as per the manufacturer's protocol. The results were collected using the Zeiss fluorescence photomicroscope and quantified by counting 10 random fields. Annexin V‐propidium iodide staining and flow cytometric analyses were completed using a FITC Annexin V Apoptosis Detection Kit (BD Biosciences, San Jose, CA, USA) on a FACS Calibur (BD Biosciences). The cells were treated with 25 ng·mL^−1^ doxorubicin (Selleck) for 24 h before analysis by flow cytometry.

#### 2.3.3 Xenograft studies

In this study, a subcutaneous xenograft mouse model was used to observe the bioactivities of cells *in vivo*. We injected 2 × 10^6^ cells (HCT‐116 and NCM460, with normal expression or overexpression of LNRRIL6) in 100 μL of phosphate‐buffered saline mixed with 30% Matrigel into the flanks of athymic nude mice (*n* = 5 per group; SLRC Laboratory Animal Center). Subcutaneous tumor volumes were evaluated every 7 days using caliper measurements for a total of 28 days. Tumor volumes were calculated using the formula *V* = 0.5 × *L* × *W* ^2^ (*L*, length and *W*, width). Twenty‐eight days after injection, all mice were sacrificed with chloral hydrate (400 mg·kg^−1^), and subcutaneous tumors were resected and weighed. For immunohistochemical studies, xenograft tumors were fixed in 10% neutral‐buffered formalin, embedded in paraffin, deparaffinized, and rehydrated, followed by antigen retrieval to evaluate proliferation and apoptosis in tumor tissues. Next, the sections were incubated with antibodies against Ki‐67 for cellular proliferation (Abcam, Hong Kong, China) or cleaved caspase‐3 for cellular apoptosis (Cell Signaling Technology, Boston, MA, USA), followed by incubation with horseradish peroxidase‐conjugated secondary antibody (Invitrogen). All slides were visualized with 3,3‐diaminobenzidine and observed using a Zeiss fluorescence photomicroscope. Experiments were also performed in LNRRIL6 knockdown (sh1 and sh2) cells.

### 2.4 Elucidating the mechanisms associated with the IL‐6−STAT3 pathway

#### 2.4.1 Isolation of cytoplasmic and nuclear RNA

First, the subcellular location of LNRRIL6 in CRC cells was determined to explore the mechanisms underlying the oncogenic role of LNRRIL6 in CRC. Then, cytoplasmic and nuclear RNA was isolated and purified with the Cytoplasmic and Nuclear RNA Purification Kit (Norgen, Belmont, CA, USA) as per the manufacturer's instructions. The isolated RNA was assessed by qRT–PCR, as described earlier.

#### 2.4.2 Chromatin isolation using the RNA purification assay

Chromatin isolation was performed using RNA purification (ChIRP) assays with the antisense probe setting against LNRRIL6 or LacZ (negative control) to determine whether LNRRIL6 binds to the IL‐6 promoter region. The ChIRP assay was performed using the Magna ChIRP RNA Interactome Kit (Millipore, Bedford, MA, USA) as per the manufacturer's instructions. Antisense DNA probes against LNRRIL6 were designed by Biosearch Probe Designer. The probe sequences used were as follows: 1, 5′‐CAGCCCTGAAAATGTGTGAC‐3′; 2, 5′‐AGAATGAATCATCTGGGGCC‐3′; 3, 5′‐CTTTGGGTTTCTAACCTGAG‐3′; 4, 5′‐CACAAACTCCTACCCAAACT‐3′; 5, 5′‐TGGAATCACTTGTTGCTGTC‐3′; 6, 5′‐GGCATGAGCAAAGGACTTTG‐3′; 7, 5′‐AGCATGAATCCTGAGGTTTT‐3′; 8, 5′‐GTAAGGGGAGTCAGGAGGAG‐3′; 9, 5′‐GGCGGAGACTGAGTCATTAA‐3′; and 10, 5′‐AATTCTCAGACACTCTCAGC‐3′. Isolated DNA was then used for qRT–PCR analysis to assess the enrichment of chromatin. The gene‐specific primer sequences used were as follows: IL‐6 promoter, 5′‐ATTGCTTGAACCTGGGAG‐3′ (forward) and 5′‐ATTTGCTTGTGGGAGAGATG‐3′ (reverse); STAT3 promoter, 5′‐CCCATGTTCTTTTTGTTGTCC‐3′ (forward) and 5′‐GAGGTTGAGAGCCTCTTACC‐3′ (reverse); and β‐actin promoter, 5′‐CAGACATACAACGGACGG‐3′ (forward) and 5′‐GTGATGAAGGCTACAAACC‐3′ (reverse).

#### 2.4.3 Assessing IL‐6 levels and IL‐6 mRNA expression

The enzyme‐linked immunosorbent assay (ELISA) was used to assess IL‐6 levels in the supernatant of the culture medium 48 h after the cells were detected (Human IL‐6 ELISA Kit; Dakewe Biotech Company, Shanghai, China) as per the manufacturer's protocol. IL‐6 mRNA expression was evaluated by qRT–PCR as previously described. These experiments were repeated for both LNRRIL6 overexpressing and LNRRIL6 knockdown cells (sh1 and sh2).

#### 2.4.4 Western blot analysis

Western blot analysis was performed to evaluate the phosphorylation levels of STAT1, STAT2, STAT 3, STAT5, and STAT6. Total proteins were retrieved from HCT‐116 cells using RIPA buffer (Beyotime, Jiangsu, China). Equal amounts of protein were loaded onto 10% sodium dodecyl sulfate/polyacrylamide gels and separated by electrophoresis, followed by transfer to polyvinylidene fluoride membranes. After blocking with 5% bovine serum albumin, membranes were incubated with primary antibodies against normal and phosphorylated STAT1, STAT2, STAT3, STAT5, and STAT6 (Cell Signaling Technology) and β‐actin (Proteintech, Rosemont, IL, USA). After washing, the membranes were incubated with IRDye 800 CW goat anti‐rabbit IgG or IRDye 700 CW goat anti‐mouse IgG (Li‐Cor, Lincoln, NE, USA), and protein levels were detected using an Odyssey infrared scanner (Li‐Cor). All experiments were repeated for LNRRIL6 overexpressing and LNRRIL6 knockdown cells (sh1 and sh2).

#### 2.4.5 Validation using an IL‐6 receptor antagonist, tocilizumab

Tocilizumab (5 μg·mL^−1^), an antagonist of the IL‐6 receptor, was used as an inhibitor of the IL‐6 signaling pathway to confirm the role of the IL‐6 pathway in the oncogenic mechanisms of LNRRIL6. In addition, we performed the Glo cell viability assay, EdU assay, and TUNEL assay to assess the effects of LNRRIL6 on cell viability, proliferation, and apoptosis after exposure to tocilizumab in LNRRIL6 overexpressing HCT‐116 cells.

### 2.5 Statistical analysis

In this study, statistical analyses were performed using spss software (V 20.0.0., IBM, Chicago, IL, USA). Comparisons between the groups were performed according to the normal distribution by using two‐way analysis of variance (ANOVA) followed by Bonferroni post hoc correction. Data that were not normally distributed (LNRRIL6 expression in 66 pairs of CRC tissues and adjacent normal mucosal tissue) were subjected to the Wilcoxon signed‐rank test, and data from the Kaplan–Meier survival analysis were subjected to the log‐rank test. In addition, we performed Pearson chi‐square test for comparing the rate. Data with normal distribution and homoscedasticity were analyzed with the Student's *t*‐test (quantitative analyses for the EdU and TUNEL assays). Furthermore, nonhomogeneous data with normal distribution were analyzed with the Mann–Whitney *U*‐test. The correlation between IL‐6 and LNRRIL6 expression levels in 66 CRC tissues was assessed by Pearson's correlation analysis. *P* < 0.05 was considered statistically significant.

## 3 Results

### 3.1 LNRRIL6 expression in tissues and patients with CRC

**Figure 1:**
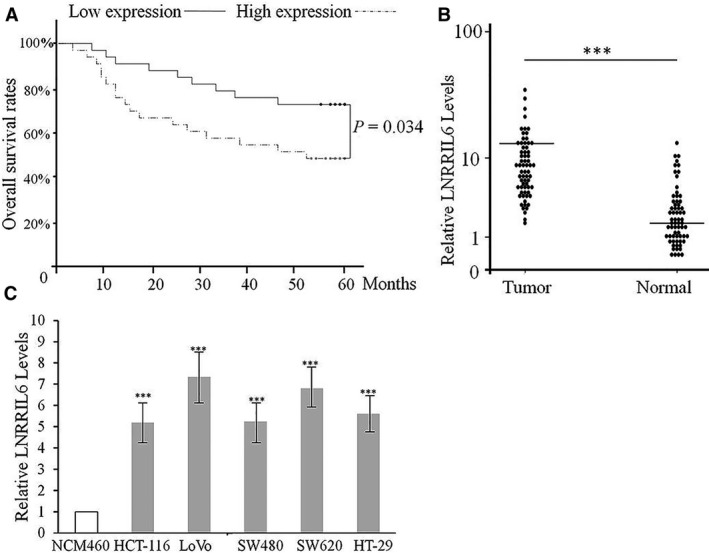
The relationship between LNRRIL6 expression and clinical outcomes in patients with CRC. (A) Kaplan–Meier survival curve showing that patients with low expression of LNRRIL6 exhibit a better outcome. (B) Distribution of LNRRIL6 in CRC tissues and normal control. LNRRIL6 expression in tumor tissues is significantly higher than in normal mucosal tissue. C, All CRC cell lines, including HCT‐116, LoVo, SW480, SW620, and HT‐29, exhibit higher expression of LNRRIL6. Data are expressed as mean ± standard deviation (average of three replicated experiments); Kaplan–Meier survival analysis was analyzed with log‐rank test, here Pearson chi‐square test was also performed for comparing the rate.; distribution of LNRRIL6 was analyzed with Wilcoxon signed‐rank test; comparisons of the relative LNRRIL6 levels between the groups were analyzed with ANOVA followed by *post hoc* correction; ***P < 0.001, NCM460 is the normal control cell line.

The findings of this study demonstrated that patients with higher expression of LNRRIL6 might present with larger tumor sizes, poor differentiation, and increased potential for lymph node metastasis (*P* < 0.05; Table 1). The Kaplan–Meier survival analysis revealed that lower expression of LNRRIL6 resulted in better outcomes (mean: 49 months vs. 36 months; *P* < 0.01; Fig. 1A). Clinically, our data suggest that the expression of LNRRIL6 is closely related to clinical outcome, whereby upregulation of LNRRIL6 is associated with worse outcomes. Similar results were obtained from the analysis of tissues from pathological specimens. Thus, LNRRIL6 is significantly upregulated in tumor tissues (*P* < 0.01; Fig. 1B). In addition, all tested CRC cells exhibited a higher expression of LNRRIL6 compared with the normal control cell line (normal epithelial cell line, *P* < 0.01; Fig. 1C). Furthermore, clinical, tissular, and cellular evidence suggests that LNRRIL6 is associated with worsened CRC prognosis.

### 3.2 Correlation between LNRRIL6 and the proliferation and survival of CRC cells

This study validated the effects of LNRRIL6 on CRC cells *in vitro* and *in vivo* by directly observing LNRRIL6 overexpressing and LNRRIL6 knockdown cells.

#### 3.2.1 Validation *in vitro*

**Figure 2:**
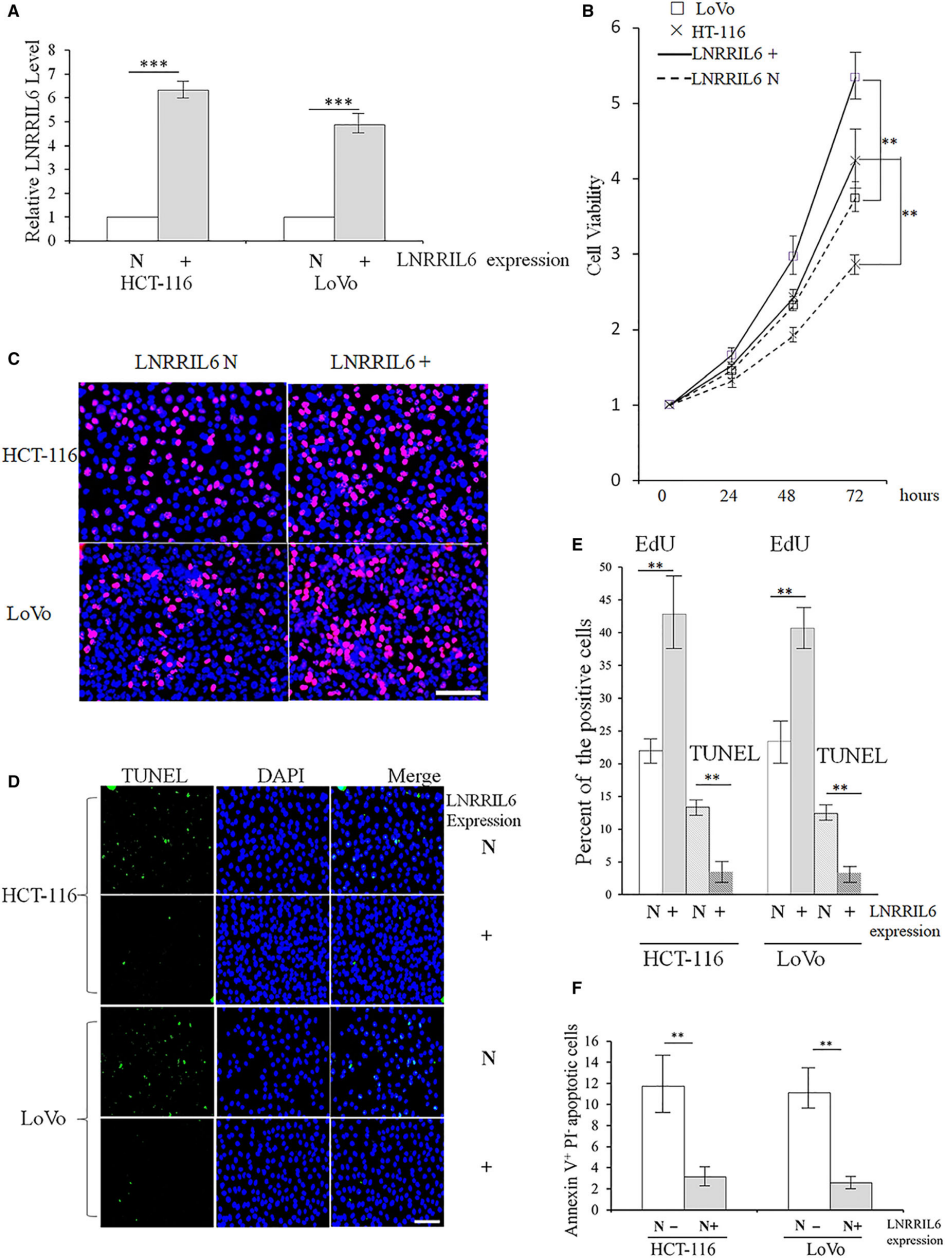
LNRRIL6 enhances the proliferation and cell survival of CRC cells *in vitro*. (A) Confirmation of LNRRIL6 expression in HCT‐116^+^ and LoVo^+^ cell lines. Upregulation of LNRRIL6 was confirmed in LNRRIL6^+^ cells. (B) Viability of LNRRIL6^+^ cells significantly increased in both HCT‐116^+^ and LoVo^+^ cell lines. (C) Representative images of EdU staining; red cells indicate EdU‐positive cells. In both cell lines, there were more red cells in the LNRRIL6^+^ group than in the LNRRIL6 N group. (D) Representative images of TUNEL staining; green cells indicate TUNEL‐positive cells. In both cell lines, there were fewer green cells in the LNRRIL6^+^ group compared with the LNRRIL6 N group. (E) Quantification of EdU and TUNEL staining. Results for HCT‐116 and LoVo are similar. Positive cells in the LNRRIL6^+^ groups were significantly increased following EdU staining and were significantly reduced following TUNEL staining. (F) Results obtained from Annexin V‐propidium iodide staining and flow cytometric analyses in both HCT‐116^+^ and LoVo^+^ cell lines were similar to those obtained from TUNEL staining. LNRRIL6^+^ indicates LNRRIL6 overexpression; LNRRIL6 N indicates LNRRIL6 normal expression; data are expressed as mean ± standard deviation (average of three replicated experiments); they were analyzed with ANOVA followed by post hoc correction; **P < 0.01; ***P < 0.001; scale bar, 100 μm.

We observed the effects of LNRRIL6 in LNRRIL6 overexpressing (LNRRIL6^+^) HCT‐116 and LoVo cells. Figure 2A confirms the successful establishment of LNRRIL6 overexpression in HCT‐116 and LoVo cell lines. After assessing cell viability at 24, 48, and 72 h, LNRRIL6^+^ cells demonstrated significantly higher viability in both HCT‐116 and LoVo cell lines (Fig. 2B). EdU staining revealed significantly more EdU‐positive cells in the LNRRIL6^+^ group, indicating higher cellular proliferation compared with the LNRRIL6 normal‐expressing (LNRRIL6 N) group (Fig. 2C,E). In addition, TUNEL assays revealed that the LNRRIL6^+^ group had fewer TUNEL‐positive cells, suggesting that this group exhibits less apoptosis than the LNRRIL6 N group (Fig. 2D,E). Moreover, Annexin V‐propidium iodide staining and flow cytometric analyses also revealed that the LNRRIL6+ group had fewer apoptotic cells (Fig. 2F). Similar findings were obtained for NCM460 cells (Fig. S3A–G).

**Figure 3:**
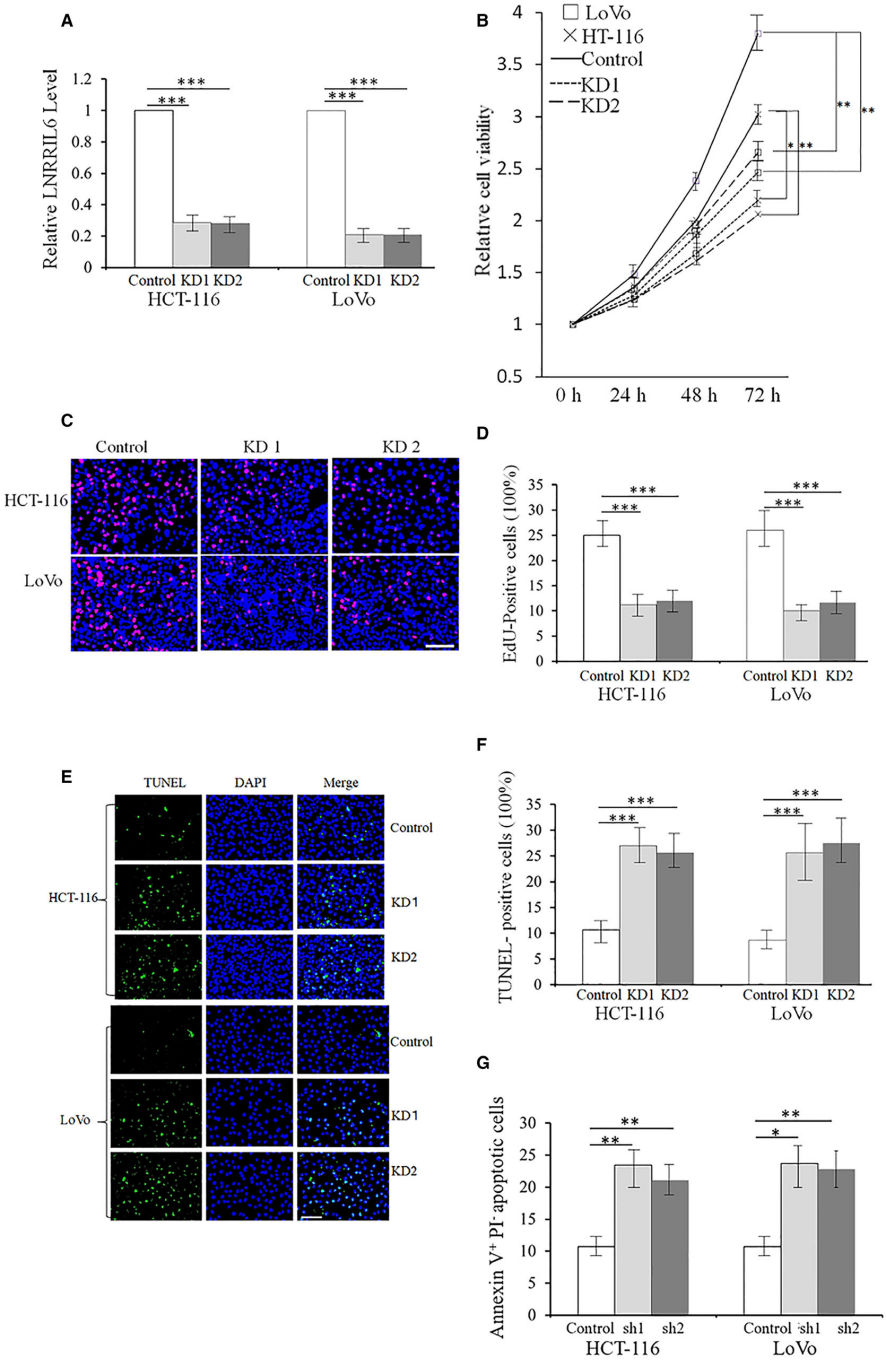
The knockdown of LNRRIL6 decreases proliferation and cell survival of CRC cells *in vitro*. (A) Confirmation of the knockdown processes. Our data confirmed notable effects of the knockdown. Both knockdown processes significantly decreased the expression of LNRRIL6 in HCT‐116 and LoVo cell lines. (B) Knockdown of LNRRIL6 significantly reduced the viability of HCT‐116 and LoVo cells. (C) Representative images of EdU staining; red cells indicate EdU‐positive cells. Number of red cells in the sh1 and sh2 groups was reduced in both HCT‐116 and LoVo cell lines. (D) Quantification of EdU staining. Both knockdown processes significantly reduced EdU‐positive cells in both cell lines. (E) Representative images of TUNEL staining; green cells indicate TUNEL‐positive cells. Number of green cells in the sh1 and sh2 groups was increased in both cell lines. (F) Quantification of TUNEL staining. Both knockdown processes significantly enhanced the number of TUNEL‐positive cells. (G) Results obtained from Annexin V‐propidium iodide staining and flow cytometric analyses in both HCT‐116^+^ and LoVo^+^ cell lines were similar to those obtained from TUNEL staining. Data are expressed as mean ± standard deviation (average of three replicated experiments); they were analyzed with the Student's *t*‐test; *P < 0.05; **P < 0.01; ***P < 0.001; sh1 represents knockdown process 1; sh2 represents knockdown process 2; scale bar, 100 μm.

Interestingly, LNRRIL6 knockdown cells exhibited reverse outcomes. Fig. 3A confirms the successful establishment of LNRRIL6 knockdown in both HCT‐116 and LoVo cell lines. In addition, cell viability analysis at 24, 48, and 72 h revealed that knockdown of LNRRIL6 significantly reduced the viability in both HCT‐116 and LoVo cell lines (Fig. 3B). EdU staining demonstrated that knockdown of LNRRIL6 resulted in fewer EdU‐positive cells, suggesting that LNRRIL6 knockdown decreases cellular proliferation compared with LNRRIL6 normal cells (Fig. 3C,D). In addition, TUNEL assays revealed that knockdown of LNRRIL6 led to increased numbers of TUNEL‐positive cells, suggesting that knockdown of LNRRIL6 induces a higher degree of apoptosis compared to cells with normal expression of LNRRIL6 (Fig. 3E,F). Similar results were obtained from Annexin V‐propidium iodide staining and flow cytometric analyses, whereby knockdown of LNRRIL6 resulted in increased proportions of apoptotic cells (Fig. 3G). Hence, this study provides support for the protective effects of LNRRIL6 on CRC cells *in vitro*.

#### 3.2.2 Validation *in vivo*

**Figure 4:**
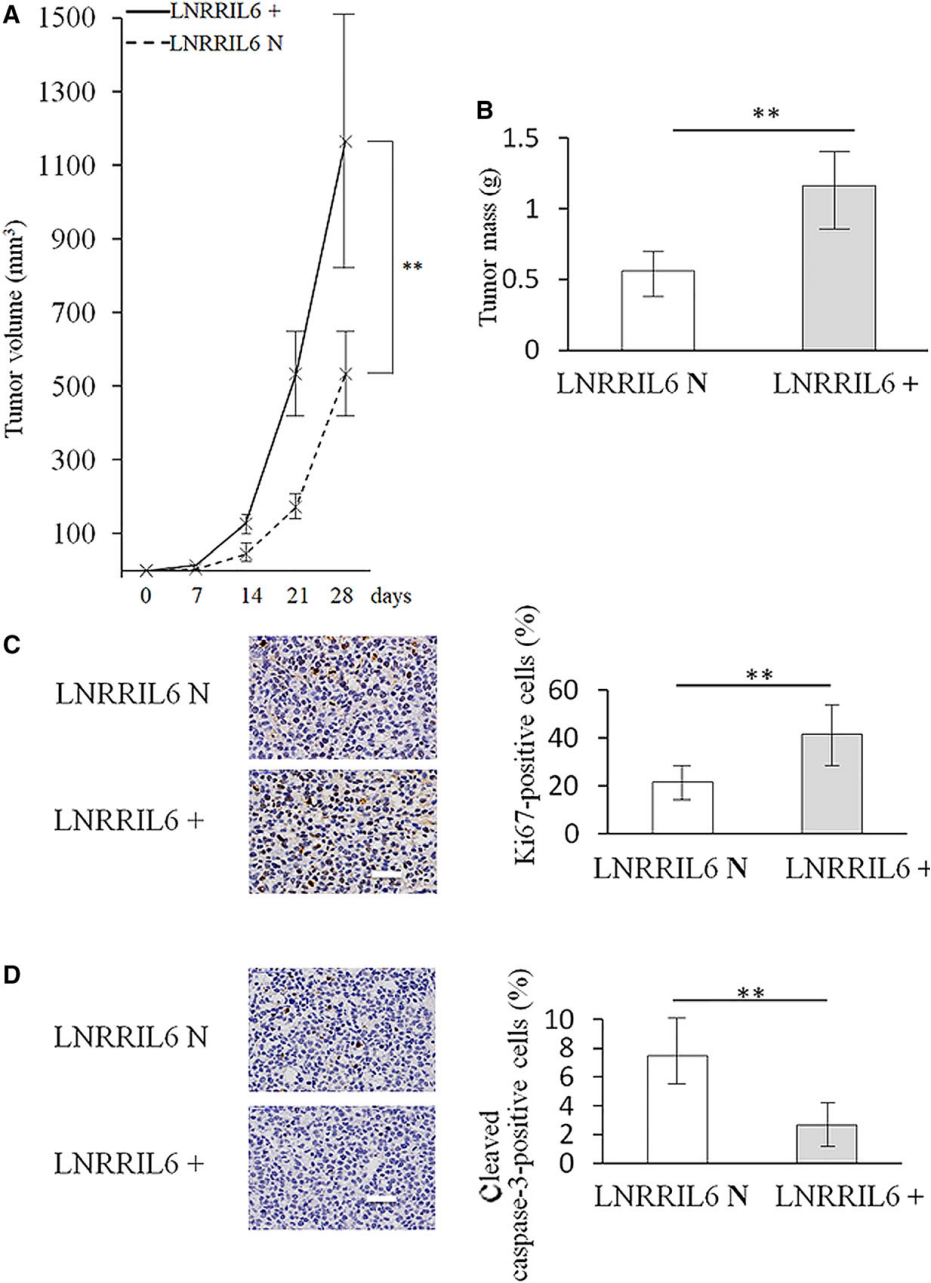
Validation of the effect of LNRRIL6 on HCT‐116 cells *in vivo*. (A) Changes in tumor volume of mice inoculated with LNRRIL6‐overexpressing HCT‐116 cells (LNRRIL6^+^) and LNRRIL6 normal‐expressing HCT‐116 cells (LNRRIL N). Tumors comprised of LNRRIL6^+^ cells exhibited increased volume. (B) Changes in the tumor weight of mice inoculated with LNRRIL6^+^ and LNRRIL6 N cells. Tumors comprised of LNRRIL6^+^ cells exhibited increased tumor size. (C) Left column, representative images of Ki‐67 staining; yellow cells indicate Ki‐67‐positive cells. Right column, quantification of Ki‐67 staining. The number of Ki‐67‐positive cells in the LNRRIL6^+^ cell line was significantly greater than in the LNRRIL6 N cell line. (D) Left column, representative images of cleaved caspase‐3 staining; yellow cells indicate cleaved caspase‐3‐positive cells. Right column, quantification of cleaved caspase‐3 staining. The number of cleaved caspase‐3‐positive cells in the LNRRIL6^+^ cell line was significantly less than in the LNRRIL6 N cell line. LNRRIL6^+^ indicates LNRRIL6 overexpression; LBRRIL6 N indicates LNRRIL6 normal expression; data are expressed as mean ± standard deviation (average of three replicated experiments); they were analyzed with Student's *t*‐test; **P < 0.01; scale bar, 100 μm.

To validate the effects of LNRRIL6 *in vivo*, we observed LNRRIL6 overexpressing and knockdown cells after injection into nude mice. Our findings revealed that tumor volume (Fig. 4A) and mass (Fig. 4B) of mice inoculated with LNRRIL6 overexpressing cells were significantly higher than those of mice with LNRRIL6 normal‐expressing cells. Analogously, the LNRRIL6^+^ group exhibited greater numbers of Ki‐67‐positive cells, suggesting higher rates of proliferation (Fig. 4C). In addition, the LNRRIL6^+^ group exhibited fewer cleaved caspase‐3‐positive cells, indicating less death of CRC cells in the LNRRIL6^+^ group (Fig. 4D). Similar findings were observed using NCM460 cells. Although NCM460 cells displayed limited tumorigenicity, the volume and mass of the tumors containing LNRRIL6^+^ cells were significantly higher than those obtained from LNRRIL N cells (Fig. S3H,I).

**Figure 5:**
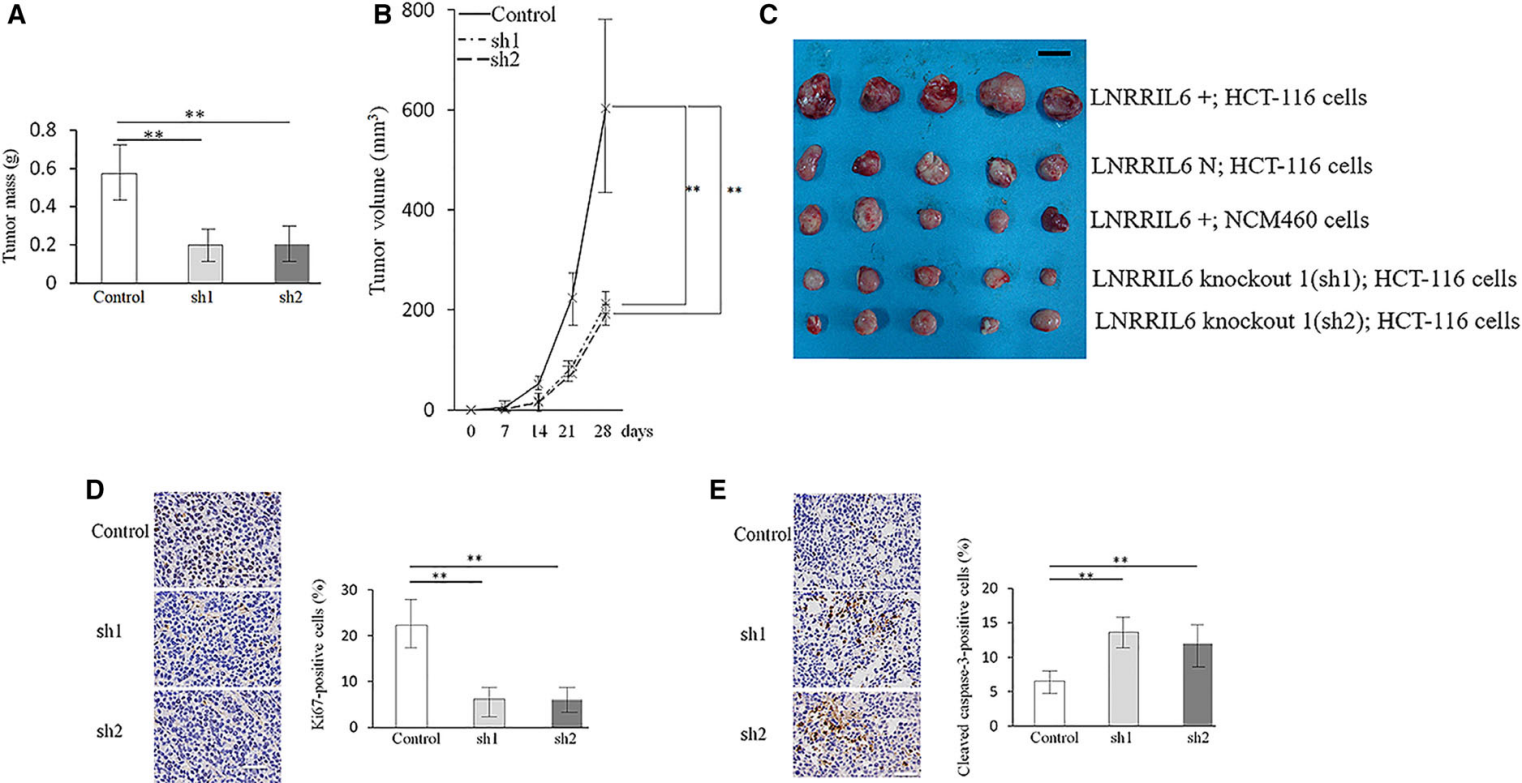
Validation of the effect of LNRRIL6 knockdown on HCT‐116 cells *in vivo*. (A) Changes in tumor volume induced by the knockdown of LNRRIL6. Knockdown of LNRRIL6 significantly reduced tumor volume in mice inoculated with HCT‐116 cells. (B) Changes in tumor weight caused by knockdown of LNRRIL6. Knockdown of LNRRIL6 significantly reduced the tumor weight of mice. (C) Representative macroscopic images of the tumors derived from injection of mice with LNRRIL6^+^, LNRRIL6 knockdown, and control cells. The black bar = 1 cm. (D) left column, representative images of Ki‐67 staining; right column, quantification of Ki‐67 staining. The number of Ki‐67‐positive cells was significantly reduced by the knockdown of LNRRIL6 in HCT‐116 cells. (E) Left column, representative images of cleaved caspase‐3 staining; right column, quantification of cleaved caspase‐3 staining. The number of cleaved caspase‐3‐positive cells was significantly increased by the knockdown of LNRRIL6. Data are expressed as mean ± standard deviation (average of three replicated experiments); *n* = 5 mice in each group; they were analyzed with ANOVA followed by post hoc correction; **P < 0.01; sh1 represents knockdown process 1; sh2 represents knockdown process 2; scale bar, 100 μm.

Opposing results were obtained from studies on LNRRIL6 knockdown cells. Tumor volume (Fig. 5A) and mass (Fig. 5B) of mice in the knockdown groups were smaller than those obtained from the LNRRIL6 normal‐expressing control group (Fig. 5C). In addition, the knockdown groups exhibited fewer Ki‐67‐positive cells (Fig. 5D) and greater cleaved caspase‐3‐positive cells (Fig. 5E), suggesting less proliferation and more cell death in LNRRIL6‐knockdown cells.

Based on *in vitro* and *in vivo* validation, as well as evidence from the overexpression and knockdown of LNRRIL6 cells, this study significantly establishes the cellular effects of LNRRIL6 on CRC cells.

### 3.3 Elucidating mechanisms associated with the IL‐6–STAT3 pathway

**Figure 6:**
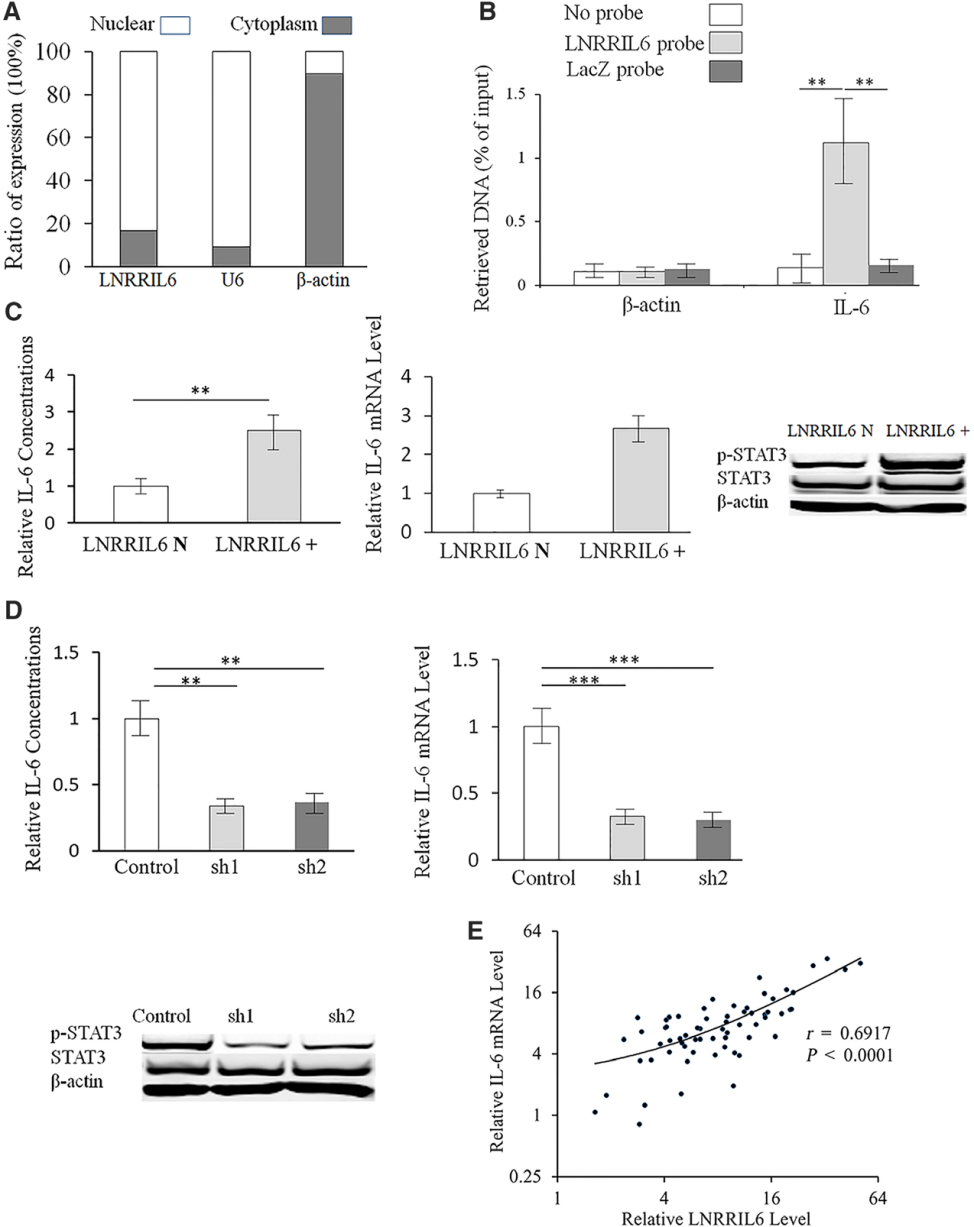
LNRRIL6 binds to the IL‐6 promoter and activates the IL‐6–STAT3 pathway. (A) Compared with controls (U6 for nuclear and β‐actin for cytoplasm), we found that the subcellular location of LNRRIL6 is mainly distributed in the nucleus. (B) Results of the ChIRP assay. The LNRRIL6 antisense probe pull‐down group exhibited significant enrichment only in the IL‐6 promoter region (right column) (*P* < 0.01) compared with negative control (LacZ) and blank control (no probe) by using ANOVA followed by post hoc correction. Enrichment was not detected in the β‐actin region. These data indicate that LNRRIL6 directly binds to the IL‐6 promoter region. (C) Results of ELISA revealed a significant enhancement of IL‐6 concentration in cell supernatants of LNRRIL6‐overexpressing (LNRRIL6^+^) HCT‐116 cells (left) compared with LNRRIL6 normal‐expressing (LNRRIL6 N) HCT‐116 cells. In addition, the expression of IL‐6 mRNA was significantly upregulated in LNRRIL6^+^ cells (middle). Western blot analysis revealed that the expression of p‐STAT3 was upregulated in LNRRIL6^+^ cells (right), compared with LNRRIL6 N cells. These data were analyzed with the Mann–Whitney *U*‐test. (D) IL‐6 concentration (left), expression of IL‐6 mRNA (middle), and expression of p‐STAT3 (right) were decreased by LNRRIL6 knockdown (sh1 and sh2) in HCT‐116 cells. These data were analyzed with the Mann–Whitney U‐test. (E) There was a positive correlation between the IL‐6 mRNA level and the LNRRIL6 expression level in CRC tissues. These data were analyzed by using Pearson's correlation analysis. LNRRIL6^+^ indicates LNRRIL6 overexpression; LBRRIL6 N indicates LNRRIL6 normal expression; data are expressed as mean ± standard deviation (average of three replicated experiments); **P < 0.01; ***P < 0.001; sh1 represents knockdown process 1; sh2 represents knockdown process 2.

Figure 6A confirms that LNRRIL6 was primarily localized in the nucleus. Results from the ChIRP assay demonstrated that the LNRRIL6 antisense probe pull‐down group exhibited significant enrichment only in the IL‐6 promoter region (LacZ; *P* < 0.01). We observed no enrichment effect in the β‐actin promoter region. Moreover, the LNRRIL6 antisense probe pull‐down group did not exhibit enrichment effects in the STAT3 promoter region (Fig. S4A). These findings suggest that LNRRIL6 directly binds to the IL‐6 promoter region (Fig. 6B). Using an LNRRIL6 overexpression (LNRRIL6^+^) cell model, we established that IL‐6 levels in cell supernatants were significantly higher than those in LNRRIL6 normal expression (LNRRIL N) cells. In addition, IL‐6 mRNA was significantly upregulated in CRC tissues, and expression of p‐STAT3 was remarkably upregulated in LNRRIL6^+^ cells (Fig. 6C). The expression of p‐STAT1, p‐STAT2, p‐STAT5, and p‐STAT6 was unchanged in LNRRIL6^+^ cells (Fig. S4B), whereas the expression of STAT3‐regulated genes, CDC25A, cyclin D1, survivin, and BCL2, was remarkably upregulated in LNRRIL6^+^ cells (Fig. S4C). In contrast, the knockdown of LNRRIL6 expression in cells led to significantly lower IL‐6 levels compared with the control. In addition, the expression of IL‐6 mRNA was significantly downregulated in the knockdown groups (sh1 and sh2), as was the expression of p‐STAT3 (Fig. 6D). The expression of STAT3‐regulated genes, CDC25A, cyclin D1, survivin, and BCL2, were remarkably downregulated in the knockdown groups (Fig. S4D). Thus, IL‐6 mRNA level and LNRRIL6 expression exhibited a positive association in CRC tissues (*r* = 0.6917; *P* < 0.001; Fig. 6E). Based on these findings, it can be surmised that the IL‐6–STAT3 pathway might play a role in the protumoral mechanisms of LNRRIL6.

**Figure 7:**
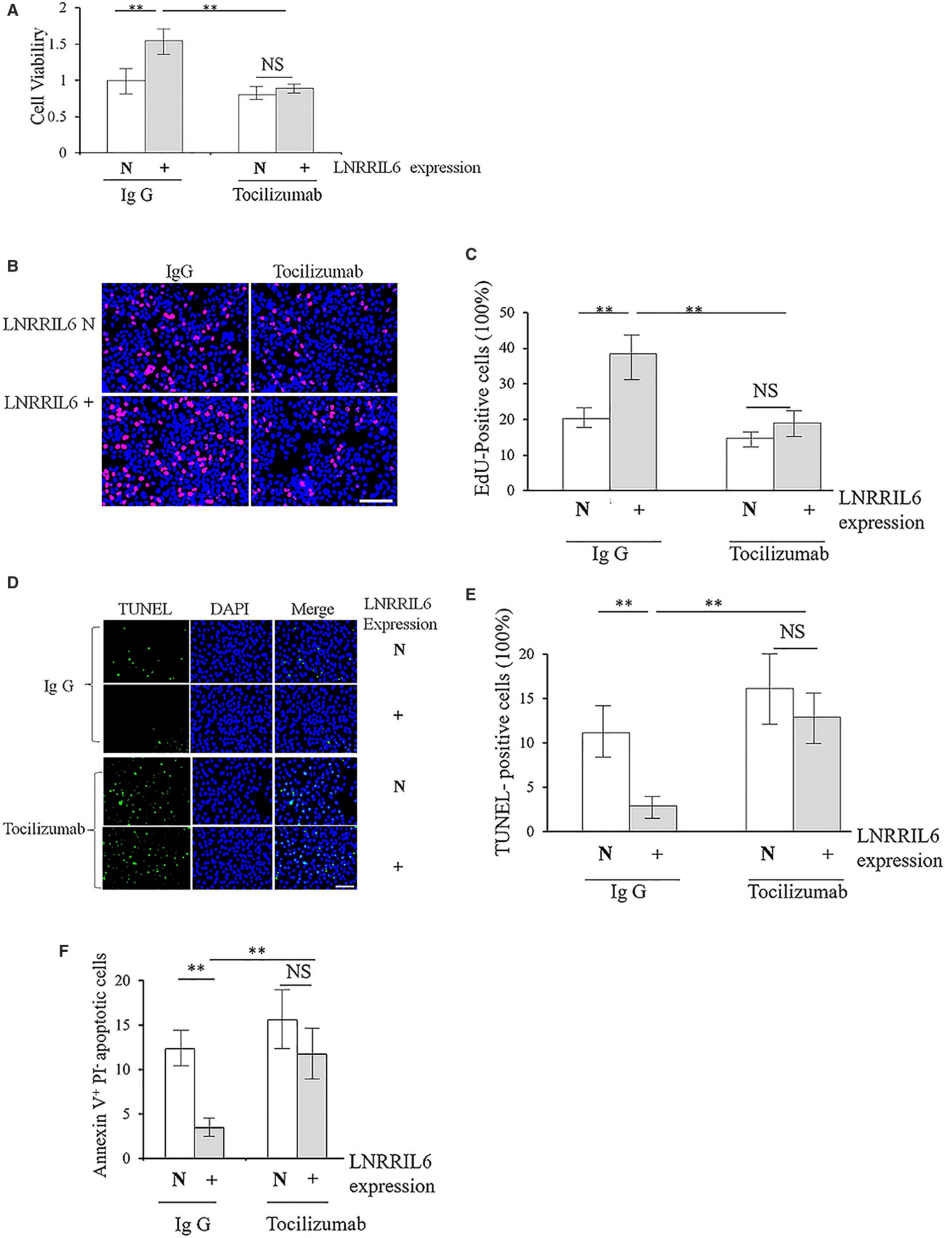
Validation of the IL‐6–STAT3 pathway‐related mechanism using the IL‐6 receptor antagonist, tocilizumab, in HCT‐116 cells. (A) Results of the Glo cell viability assay indicate that in the presence of IgG, the LNRRIL6‐overexpressing (LNRRIL6^+^) HCT‐116 cells exhibited significantly increased the viability (*P* < 0.01), whereas administration of tocilizumab abrogates this effect. No significant difference was observed between the LNRRIL6^+^ and LNRRIL6 normal‐expressing (LNRRIL6 N) HCT‐116 cells. (B) Representative images of EdU staining; red cells indicate EdU‐positive cells. In the presence of IgG, there were an increased number of red cells in the LNRRIL6^+^ group compared with the LNRRIL6 N group. However, administration of tocilizumab abrogated this effect. (C) Quantification of EdU staining. In the presence of IgG, the number of EdU‐positive cells in the LNRRIL6^+^ groups was significantly higher than those in the LNRRIL6 N groups (*P* < 0.01); however, administration of tocilizumab reduced this difference. (D) Representative images of TUNEL staining; green cells indicate the TUNEL‐positive cells. In the presence of IgG, there were fewer green cells in the LNRRIL6^+^ group compared with the LNRRIL6 N group. However, administration of tocilizumab reduced this difference. (E) Quantification of TUNEL staining. In the presence of IgG, the number of TUNEL‐positive cells in the LNRRIL6^+^ groups was significantly lower than those in the LNRRIL6 N groups (*P* < 0.01); however, administration of tocilizumab reduced this difference. (F) Results obtained from Annexin V‐propidium iodide staining and flow cytometric analyses were similar to those obtained from TUNEL staining. LNRRIL6^+^ indicates LNRRIL6 overexpression; LBRRIL6 N indicates LNRRIL6 normal expression; data are expressed as mean ± standard deviation (average of three replicated experiments); they were analyzed with ANOVA followed by *post hoc* correction; **P < 0.01; scale bar, 100 μm.

The IL‐6 receptor antagonist, tocilizumab, was used to obtain concrete evidence of possible IL‐6‐related mechanisms. Results revealed that tocilizumab significantly reduced cell viability in both LNRRIL6^+^ and LNRRIL6 N cells (Fig. 7A). EdU staining data suggested that tocilizumab may reduce the LNRRIL6^+^‐induced proliferation of CRC (Fig. 7B,C). In addition, TUNEL staining results, along with Annexin V‐propidium iodide staining and flow cytometric analyses, demonstrated that tocilizumab enhanced apoptosis in CRC cells, which was reduced by the overexpression of LNRRIL6 (Fig. 7D–F). These results provide substantial evidence of the involvement of IL‐6–STAT3 pathway activation in the mechanisms responsible for the protection of CRC cells by LNRRIL6.

## 4 Discussion

This study establishes the similarity between AK024522 and the IL‐6 promoter based on the results of BLAST (Fig. S1). Hence, we defined this lncRNA as LNRRIL6 and verified the biological activities of LNRRIL6. We validated the protective effects of LNRRIL6 on CRC at human, tissular, and cellular levels. In addition, we demonstrated that these effects are correlated with IL‐6–STAT3 pathway activation. To the best of our knowledge, this is the first study to investigate the novel lncRNA, LNRRIL6 and to verify its effects in CRC. Our results are in accordance with previous studies on GPRC5A in other tumors, including head and neck squamous cell carcinoma (Liu *et al*., 2017), pancreatic cancer (Jahny *et al*., 2017), lung cancer (Zhong *et al*., 2015), and leukemia (Chen *et al*., 2010). The results of this study suggest the potential utilization of LNRRIL6 as a novel biomarker and therapeutic target for CRC, however, warrant further validation in animal models and patients.

This study provides verification of the role of LNRRIL6 from different levels. First, at a clinical level, we demonstrated that patients with high expression of LNRRIL6 exhibited a worse outcome (Fig. 1A), larger tumor size, poorer differentiation, and increased potential for lymph node metastasis (Table 1). At the tissular level, the expression of LNRRIL6 in tumor tissue is significantly higher than that in normal tissue (Fig. 1B). Finally, at the cellular level, we demonstrated higher expression of LNRRIL6 in CRC cell lines (Fig. 1C). *In vitro*, the overexpression of LNRRIL6 was associated with increased cell viability and proliferation and decreased apoptosis (Fig. 2), whereas knockdown of LNRRIL6 correlated with inhibition of cell viability and proliferation, and promotion of apoptosis (Fig. 3). Moreover, our results were strengthened by using three cell lines and generated consistent findings *in vivo*. All mice injected with LNRRIL6 overexpressing cells demonstrated larger tumors with higher rates of proliferation and lower rates of apoptosis (Fig. 4), whereas mice injected with LNRRIL6 knockdown cells exhibited opposing results, including smaller tumors, decreased proliferation, and increased apoptosis (Fig. 5). Thus, this study suggests that overexpression of LNRRIL6 may promote tumor proliferation, whereas knockdown of LNRRIL6 expression may enhance tumor apoptosis. Taken together, the results of this study provide integrated evidence of the protective capability of LNRRIL6 on CRC cells. Hence, inhibiting the expression of LNRRIL6 may negate protumoral effects and potentially benefit patients.

Due to its high consistency with the IL‐6 promoter, we considered that LNRRIL6 may act as an IL‐6 promoter through the IL‐6–STAT3 pathway. The roles of IL‐6 in the pathogenesis of cancer are well reported. As an essential mediator of inflammatory responses and activator of STAT3, IL‐6 could impair apoptosis in cancer cells (Hodge *et al*., 2005). Increasing evidence suggests that IL‐6 and the IL‐6–STAT3 pathways play crucial roles in the onset, development, and formation of CRC (Wang and Sun, 2014). A recent study reported that IL‐6 expression was associated with cell invasion, death, and lymph node metastasis in CRC (Zeng *et al*., 2017). In addition, the IL‐6 promoter might increase the level of IL‐6 by activation of STAT3 (Albino *et al*., 2016). This study suggests that LNRRIL6 shares similar pathological features with the IL‐6 promoter. As LNRRIL6 is primarily expressed in the nucleus, ChIRP assays revealed that LNRRIL6 directly binds to the IL‐6 promoter region. IL‐6 levels secreted in media and IL‐6 mRNA levels in tumor tissues, and expression of p‐STAT3 is upregulated in cells with overexpression of LNRRIL6 and is downregulated in LNRRIL6 knockdown cells. Additionally, LNRRIL6 expression and IL‐6 mRNA level exhibited a positive correlation in CRC tissues (Fig. 6). Furthermore, validation using tocilizumab, an IL‐6 receptor antagonist, strengthened the reliability of our evidence that LNRRIL6 acts as an IL‐6 promoter through activation of the IL‐6–STAT3 pathway (Fig. 7). The mechanisms of LNRRIL6 can be summarized as follows. We propose that LNRRIL6 acts as an IL‐6 activator, which enhances the activation of STAT3 through upregulation of IL‐6. Once LNRRIL6 is overexpressed, IL‐6 levels are consequently increased, resulting in the activation of STAT3 and STAT3‐related genes involved in cell proliferation (e.g., CDC25A, cyclin D1) or survival (e.g., survivin, BCL2). Further, activation of the IL‐6–STAT3 pathway promotes the proliferation of CRC cells and suppresses apoptosis, which leads to worse outcomes in CRC patients. Conversely, once LNRRIL6 is knocked down, the expression of IL‐6 is suppressed, and activation of STAT3 and STAT3‐related genes is reduced, resulting in the death of CRC cells, and amelioration of CRC patient outcomes. Hence, LNRRIL6 can serve as a potential biomarker and therapeutic target for CRC, whereby higher expression indicates worse outcomes. Our future studies will consider LNRRIL6 treatments in animal CRC models and human patients to assess safety and efficacy.

There are several limitations in the present study that warrant discussion. Firstly, in the tumor microenvironment, epithelial cells are not the main source of cytokines, particularly IL‐6, which is produced in large quantities by immune inflammatory cells. We did not investigate whether LNRRIL6 upregulation also occurs in immune cells. Moreover, several cytokines and factors can activate the STAT3 signaling pathway in cancer cells; thus, targeting only one of these elements may not be sufficient to suppress STAT3‐mediated oncogenic activity. Secondly, we neglected gender as a factor in the experimental design. Only female mice were used for animal experiments, whereas clinical data included both male and female patients. Several studies have identified significant differences between male and female CRC subjects (Kim *et al*., 2015; Yang *et al*., 2017); however, only a limited number of studies employed animals of both sexes. Thus, Kim *et al*. (2015) suggested the inclusion of both male and female animals for future CRC studies due to sex‐specific pathophysiological differences. Our future investigations will address and verify these limitations.

## 5 Conclusion

In summary, this study defined the novel lncRNA, LNRRIL6, and authentically verified its protective effect on CRC at clinical, tissue, and cellular levels *in vivo* using xenograft mouse models, and *in vitro* using LNRRIL6 overexpression and knockdown cell models. Results of the study suggest that LNRRIL6 may act as an IL‐6 promoter through the IL‐6–STAT3 pathway; however, its utilization as a potential biomarker and therapeutic target for CRC warrants further clinical validation.

## Conflict of interest

The authors declare no conflict of interest.

## Author contributions

JW, PC, and TA got the original ideas. JW, PC, and TA designed the study. JW, JZ, CJ, JZ collected the clinical samples. JW, JZ, CJ, JZ, HN, PC, and TA performed the experiments. JW, JZ, CJ, JZ, HN performed the data analyses. JW, PC, and TA wrote the draft. JW, JZ, CJ, JZ, HN, PC, and TA discussed and decided the final version. PC and TA supervised the study.

## Supporting information


**Fig. S1.** Searching for the putative LNRRIL6 binding locus on genomic DNA using the Basic Local Alignment Search Tool (BLAST). We identified a large number of LNRRIL6 binding loci on the IL‐6 promoter spanning 870–1180 bp of LNRRIL6.

**Fig. S2.** Experimental study design.

**Fig. S3.** Experiments using the normal epithelial cell line (NCM460). A, Confirmation of LNRRIL6 expression in a normal human colon mucosal epithelial cell line (NCM460). Cells with overexpression of LNRRIL6 were confirmed and selected for subsequent experiments. B, Viability of LNRRIL6^+^ cells was significantly increased in NCM460 cells. C, Representative images of EdU staining; red cells indicate EdU‐positive cells. There was an increase in the number of red cells in the LNRRIL6^+^ groups compared with the LNRRIL6 N groups in NCM460 cells. D, Quantitative results of EdU staining. The number of positive cells in the LNRRIL6^+^ groups were significantly increased following EdU staining. E, Representative images of TUNEL staining; green cells indicate TUNEL‐positive cells. There were fewer green cells in the LNRRIL6^+^ groups compared with the LNRRIL6 N groups in NCM460 cells. F, Quantitative results of the TUNEL staining. The number of positive cells in the LNRRIL6^+^ groups were significantly reduced following TUNEL staining. G, Cell apoptosis was also measured by Annexin V‐propidium iodide staining and flow cytometric analyses in NCM460 cells. H, Changes in the tumor volume of mice inoculated with LNRRIL6 overexpressing NCM460 cells (LNRRIL6^+^) and LNRRIL6 normal‐expressing NCM460 cells (LNRRIL N). Although the development of tumors was slower compared with CRC cell lines, all mice eventually developed tumors. Tumors comprised of LNRRIL6^+^ cells exhibited a larger volume. I, Changes in the tumor weight of mice inoculated with LNRRIL6+ and LNRRIL6 N NCM460 cells. Tumors comprised of LNRRIL6^+^ cells exhibited increased tumor size. LNRRIL6^+^ indicates LNRRIL6 overexpression; LNRRIL6 N indicates LNRRIL6 normal expression; Data are expressed as mean ± standard deviation (average of three replicated experiments); they were analyzed with the Student's t‐test; *P < 0.05; **P < 0.01; ***P < 0.001; scale bar, 100 μm.

**Fig. S4.** LNRRIL6 binds to the IL‐6 promoter and activates the IL‐6−STAT3 pathway. A, Results of the ChIRP assay. The LNRRIL6 antisense probe pull‐down group exhibited no significant enrichment in the STAT3 promoter region compared with the negative control (LacZ) and blank control (no probe). B, Western blot analysis revealed that expression of p‐STAT1, p‐STAT2, p‐STAT4, and p‐STAT5 were unchanged in LNRRIL6^+^ cells compared with LNRRIL6 N cells (HCT‐116 cell line). C, Expression of STAT3‐regulated genes, CDC25A, cyclin D1, survivin, and BCL2, were significantly upregulated in LNRRIL6^+^ cells compared with LNRRIL6 N cells (HCT‐116 cell line). D, Expression of STAT3‐regulated genes, CDC25A, cyclin D1, survivin, and BCL2, were significantly decreased following LNRRIL6 knockdown (sh1 and sh2) in HCT‐116 cells. LNRRIL6^+^ indicates LNRRIL6 overexpression; LBRRIL6 N indicates LNRRIL6 normal expression; Data are expressed as mean ± standard deviation (average of three replicated experiments); they were analyzed with ANOVA followed by post hoc correction; **P < 0.01; ***P < 0.001; sh1 represents knockdown process 1; sh2 represents knockdown process 2.
