# Supplementary figures and images for: LNRRIL6, a novel long noncoding RNA, protects colorectal cancer cells by activating the IL‐6–STAT3 pathway
Source: Mol Oncol. 2019 Sep 30;13(11):2344–60. doi: 10.1002/1878-0261.12538 (PMC6822249; doi:10.1002/1878-0261.12538)

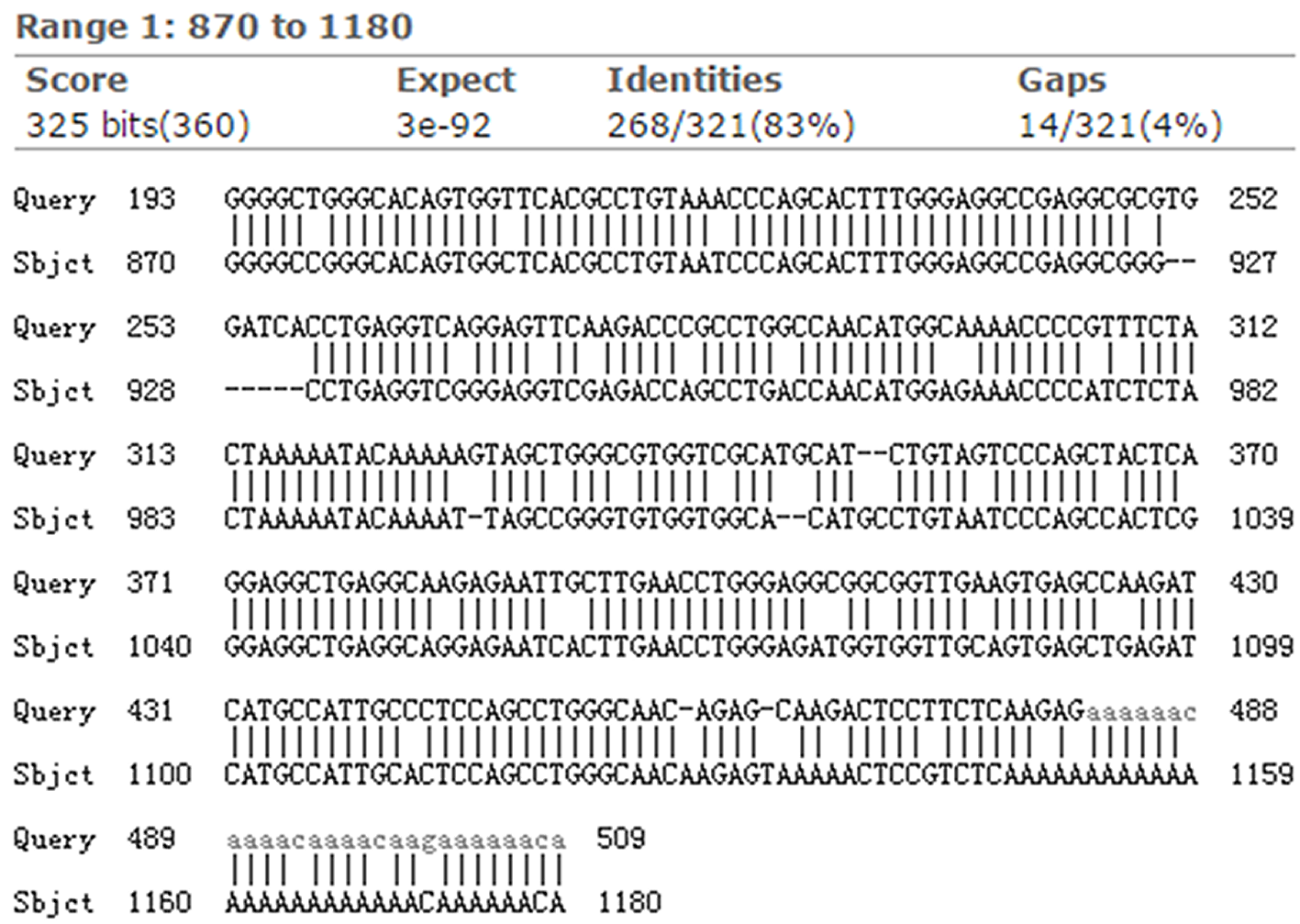

Supplement: Supplementary file 1 — Fig. S1. Searching for the putative LNRRIL6 binding locus on genomic DNA using the Basic Local Alignment Search Tool (BLAST). We identified a large number of LNRRIL6 binding loci on the IL‐6 promoter spanning 870–1180 bp of LNRRIL6. [file MOL2-13-2344-s001.tif]

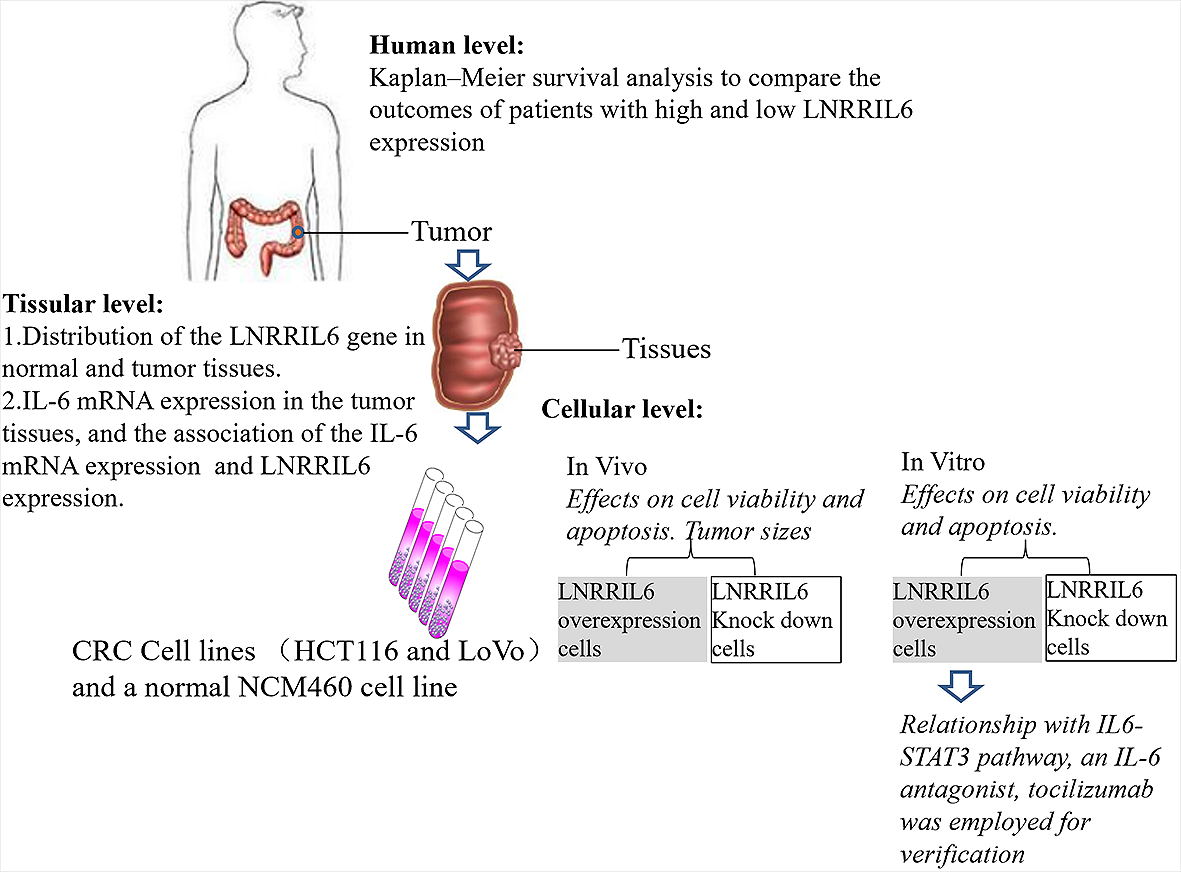

Supplement: Supplementary file 2 — Fig. S2. Experimental study design. [file MOL2-13-2344-s002.tif]

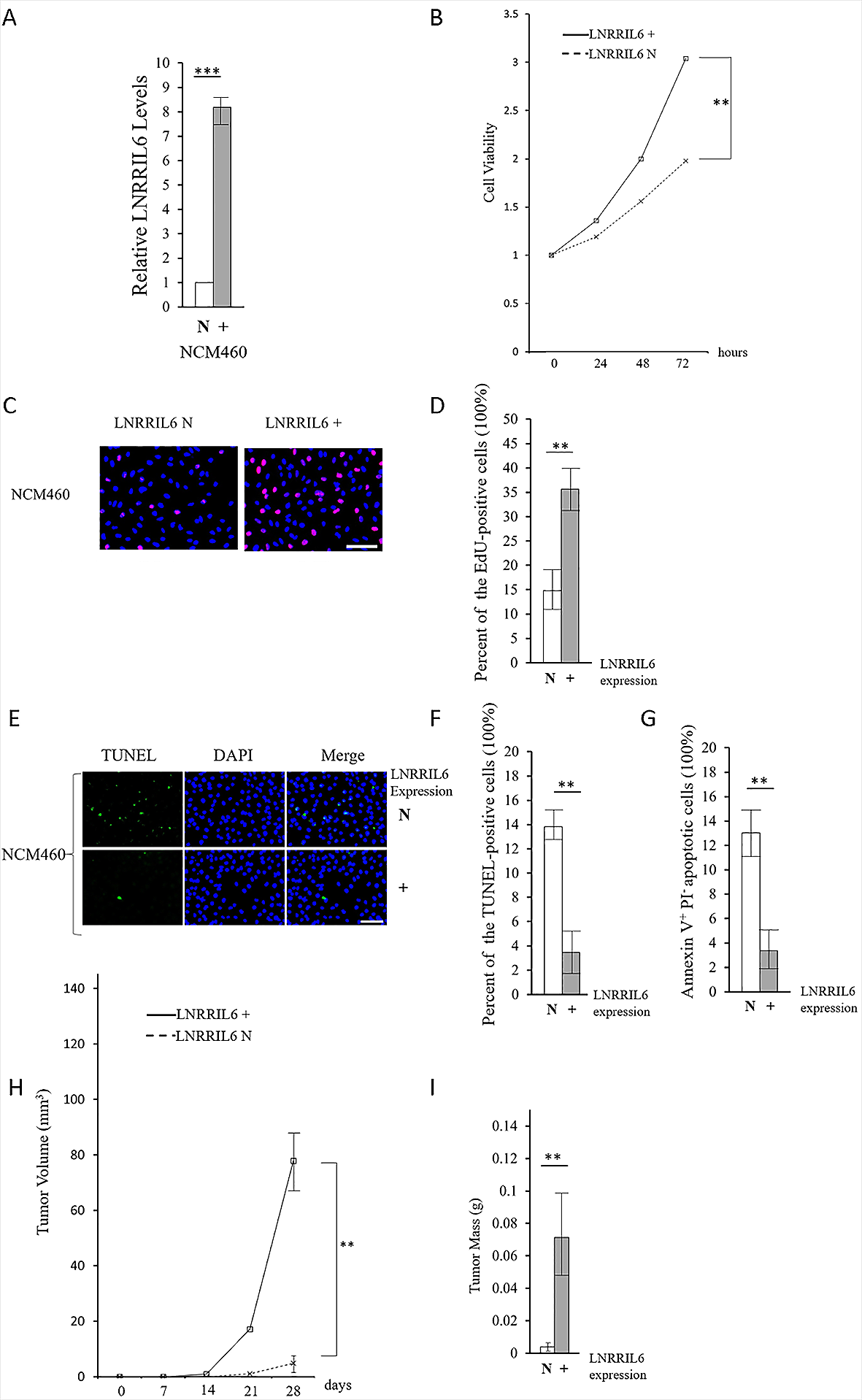

Supplement: Supplementary file 3 — Fig. S3. Experiments using the normal epithelial cell line (NCM460). A, Confirmation of LNRRIL6 expression in a normal human colon mucosal epithelial cell line (NCM460). Cells with overexpression of LNRRIL6 were confirmed and selected for subsequent experiments. B, Viability of LNRRIL6+ cells was significantly increased in NCM460 cells. C, Representative images of EdU staining; red cells indicate EdU‐positive cells. There was an increase in the number of red cells in the LNRRIL6+ groups compared with the LNRRIL6 N groups in NCM460 cells. D, Quantitative results of EdU staining. The number of positive cells in the LNRRIL6+ groups were significantly increased following EdU staining. E, Representative images of TUNEL staining; green cells indicate TUNEL‐positive cells. There were fewer green cells in the LNRRIL6+ groups compared with the LNRRIL6 N groups in NCM460 cells. F, Quantitative results of the TUNEL staining. The number of positive cells in the LNRRIL6+ groups were significantly reduced following TUNEL staining. G, Cell apoptosis was also measured by Annexin V‐propidium iodide staining and flow cytometric analyses in NCM460 cells. H, Changes in the tumor volume of mice inoculated with LNRRIL6 overexpressing NCM460 cells (LNRRIL6+) and LNRRIL6 normal‐expressing NCM460 cells (LNRRIL N). Although the development of tumors was slower compared with CRC cell lines, all mice eventually developed tumors. Tumors comprised of LNRRIL6+ cells exhibited a larger volume. I, Changes in the tumor weight of mice inoculated with LNRRIL6+ and LNRRIL6 N NCM460 cells. Tumors comprised of LNRRIL6+ cells exhibited increased tumor size. LNRRIL6+ indicates LNRRIL6 overexpression; LNRRIL6 N indicates LNRRIL6 normal expression; Data are expressed as mean ± standard deviation (average of three replicated experiments); they were analyzed with the Student's t‐test; *P < 0.05; **P < 0.01; ***P < 0.001; scale bar, 100 μm. [file MOL2-13-2344-s003.tif]

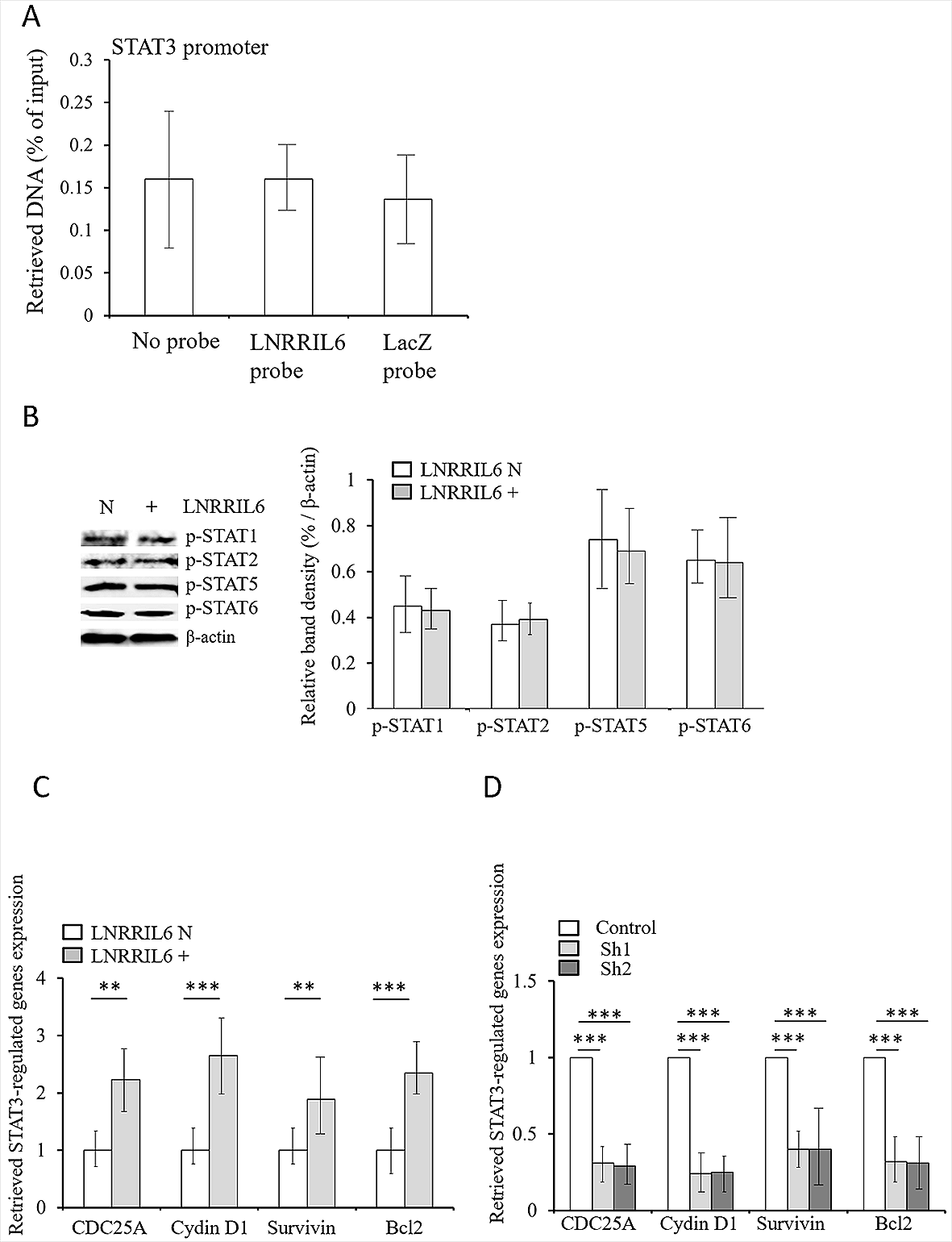

Supplement: Supplementary file 4 — Fig. S4. LNRRIL6 binds to the IL‐6 promoter and activates the IL‐6−STAT3 pathway. A, Results of the ChIRP assay. The LNRRIL6 antisense probe pull‐down group exhibited no significant enrichment in the STAT3 promoter region compared with the negative control (LacZ) and blank control (no probe). B, Western blot analysis revealed that expression of p‐STAT1, p‐STAT2, p‐STAT4, and p‐STAT5 were unchanged in LNRRIL6+ cells compared with LNRRIL6 N cells (HCT‐116 cell line). C, Expression of STAT3‐regulated genes, CDC25A, cyclin D1, survivin, and BCL2, were significantly upregulated in LNRRIL6+ cells compared with LNRRIL6 N cells (HCT‐116 cell line). D, Expression of STAT3‐regulated genes, CDC25A, cyclin D1, survivin, and BCL2, were significantly decreased following LNRRIL6 knockdown (sh1 and sh2) in HCT‐116 cells. LNRRIL6+ indicates LNRRIL6 overexpression; LBRRIL6 N indicates LNRRIL6 normal expression; Data are expressed as mean ± standard deviation (average of three replicated experiments); they were analyzed with ANOVA followed by post hoc correction; **P < 0.01; ***P < 0.001; sh1 represents knockdown process 1; sh2 represents knockdown process 2. [file MOL2-13-2344-s004.tif]
